# The informatics of ADNI

**DOI:** 10.1002/alz.14099

**Published:** 2024-08-14

**Authors:** Arthur W. Toga, Scott Neu, Sidney Taiko Sheehan, Karen Crawford, for the Alzheimer's Disease Neuroimaging Initiative

**Affiliations:** ^1^ Laboratory of Neuro Imaging (LONI) Stevens Neuroimaging and Informatics Institute Keck School of Medicine of USC University of Southern California Los Angeles California USA

**Keywords:** Alzheimer's disease, Alzheimer's Disease Neuroimaging Initiative, biomarkers, data sharing, informatics, open data

## Abstract

The Alzheimer's Disease Neuroimaging Initiative (ADNI) has revolutionized the landscape of Alzheimer's research through its Informatics Core, which has facilitated unprecedented data standardization and sharing. Over 20 years, ADNI established a robust informatics framework, enabling the validation of biomarkers and supporting global research efforts. The Informatics Core, centered at the Laboratory of Neuro Imaging (LONI), provides a comprehensive data hub that ensures data quality, accessibility, and security, fostering over 5600 publications and significant scientific advancements. By embracing open data sharing principles, ADNI set a gold standard in data transparency, allowing over 26,000 investigators from 169 countries to access and download a wealth of multimodal data. This collaborative approach not only accelerated biomarker discovery and drug development and advanced our understanding of Alzheimer's disease but also has served as a model for other research initiatives, demonstrating the transformative potential of carefully designed informatics models and shared data in driving global scientific progress.

**Highlights:**

- Accelerating biomarker discovery and drug development for Alzheimer's disease.
- Alzheimer's Disease Neuroimaging Initiative's (ADNI's) open data sharing drives scientific progress.
- Data exploration and coupled analytics to data archives.

## 1 INTRODUCTION

Celebrating 20 years of groundbreaking research, the Alzheimer's Disease Neuroimaging Initiative (ADNI) has emerged as a leader in the pursuit of optimized biomarkers for clinical trials and data transparency in the scientific community. ADNI's overarching goal has been to standardize and validate biomarkers that are crucial for Alzheimer's disease (AD) diagnosis and treatment while fostering access to data and biofluid samples worldwide. The legacy of ADNI is marked by pioneering achievements, and ADNI data have played a pivotal role in designing clinical trials for promising therapeutics. The ADNI Informatics Core, based at the Laboratory of Neuro Imaging (LONI) at the University of Southern California (USC), is responsible for de‐identifying, archiving, and disseminating all clinical, biospecimen, genetic, and imaging data including raw and processed magnetic resonance imaging (MRI) and positron emission tomography (PET) scans. All data are made available to approved ADNI investigators within days after the date of collection through the Image and Data Archive (IDA), which provides data search, exploration, and download interfaces for evaluating and obtaining data of interest. The Informatics Core also distributes methods and software tools created and/or used by the ADNI analysts.

Central to ADNI's success has been an unwavering commitment to widespread and unimpeded data sharing, which is largely facilitated by the Informatics Core. By providing access to a comprehensive and standardized data set, promoting collaboration, and enabling the development of new tools and methodologies, the ADNI Informatics Core has also played a crucial role in training scientists across various disciplines involved in AD research. ADNI data have been used extensively in training machine learning and artificial intelligence models for AD diagnosis, prognosis, and treatment development. This has provided scientists with hands‐on experience in applying cutting‐edge computational techniques to real‐world biomedical data.

**RESEARCH IN CONTEXT**

1. **Systematic review**: The authors used their accumulated knowledge and experiences from years spent working on the informatics core of the Alzheimer's Disease Neuroimaging Initiative (ADNI). The authors also reviewed the literature using traditional (e.g., PubMed) sources and relevant websites. These citations are appropriately cited.
2. **Interpretation**: The ADNI Informatics Core has established a robust informatics framework over 20 years, enabling biomarker validation and supporting global research efforts. ADNI has served as a model for other research initiatives, demonstrating the transformative potential of open data sharing in driving scientific progress.
3. **Future directions**: In this article, we propose integrating new types of biomarker data, such as voice recordings, to enhance the understanding of Alzheimer's disease progression and its functional manifestations. ADNI aims to advance data management by coupling analytics to data archives, allowing for more efficient exploration and use of large data sets.

**FIGURE 1:**
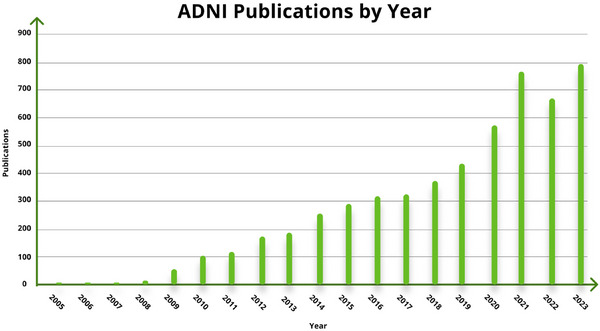
ADNI publications by year. The research output from ADNI data is not only voluminous but also diverse, covering various aspects of Alzheimer's disease, from genetic factors to biomarker development. Through 2023, the total count of publications is 5583. One of the highest‐impact ADNI publications has an RCR of 91.13, and 39 ADNI publications are in the 99th percentile or above in terms of influence, showcasing their significant contribution to the field. ADNI, Alzheimer's Disease Neuroimaging Initiative; RCR, relative citation ratio.

As of early 2024, the global literature boasts over 5600 ADNI publications (Figure 1). These milestones stand as a testament to the impact of ADNI's data sharing policy and informatics. Unlike other initiatives, ADNI's philosophy of data transparency has not hindered individual outside investigators from publishing their own findings; instead, it has catalyzed a wealth of research output from ADNI investigators utilizing shared data. By openly disseminating data, ADNI has amplified its influence on the AD and neurology fields, setting a precedent for collaborative data sharing initiatives. As we commemorate two decades of ADNI's transformative contributions, we encourage other research endeavors to embrace this ethos, recognizing the transformative potential of open data sharing in driving scientific progress forward.

The integration of large‐scale data that combines clinical, scientific, and demographic details for crafting AD therapies has gained momentum in recent times. Funding agencies have increasingly mandated data sharing in line with FAIR principles (Findable, Accessible, Interoperable, and Reusable),^1^ and some publications now require the data described in submitted manuscripts to be available in publicly accessible archives. For example, *Nature* and its associated journals requires authors to make the data available to interpret and extend the research in the manuscript.^2^ The data must be deposited in an appropriate public repository, and authors must include a statement in the manuscript on how the data can be accessed. *Science*, PLoS One, *The Lancet*, *BMJ*, *PNAS*, and other journals hold similar policies. These policies are important statements, but the ethos of open data sharing championed by ADNI since its inception in 2004 was pioneering. Back then, the idea of granting unrestricted access to all competent researchers on a global scale was groundbreaking.

Traditionally, investigators have held tight control over their data for a variety of reasons, including doubts about the trustworthiness of third‐party databases and apprehensions about how others may utilize their data. Questions have arisen about the quality of data deposited into such databases, the potential for data reuse, and the impact on future publications. Data ownership has emerged as a primary concern that hinders attempts to share data and limits availability to others. ADNI has adopted policies, procedures, and technologies to address these challenges and has set an example of how data sharing could work to everyone's benefit.

The principle of openly sharing every piece of data from a study enables the external replication of results, facilitates comprehensive meta‐analyses, and fosters the conduct of new studies using existing data sets.^3^ It even allows alternative and contradictory interpretations as to what the results show, something that ADNI embraced by encouraging peer review to adjudicate differences rather than restricting data access or imposing publication committee control.

**FIGURE 2:**
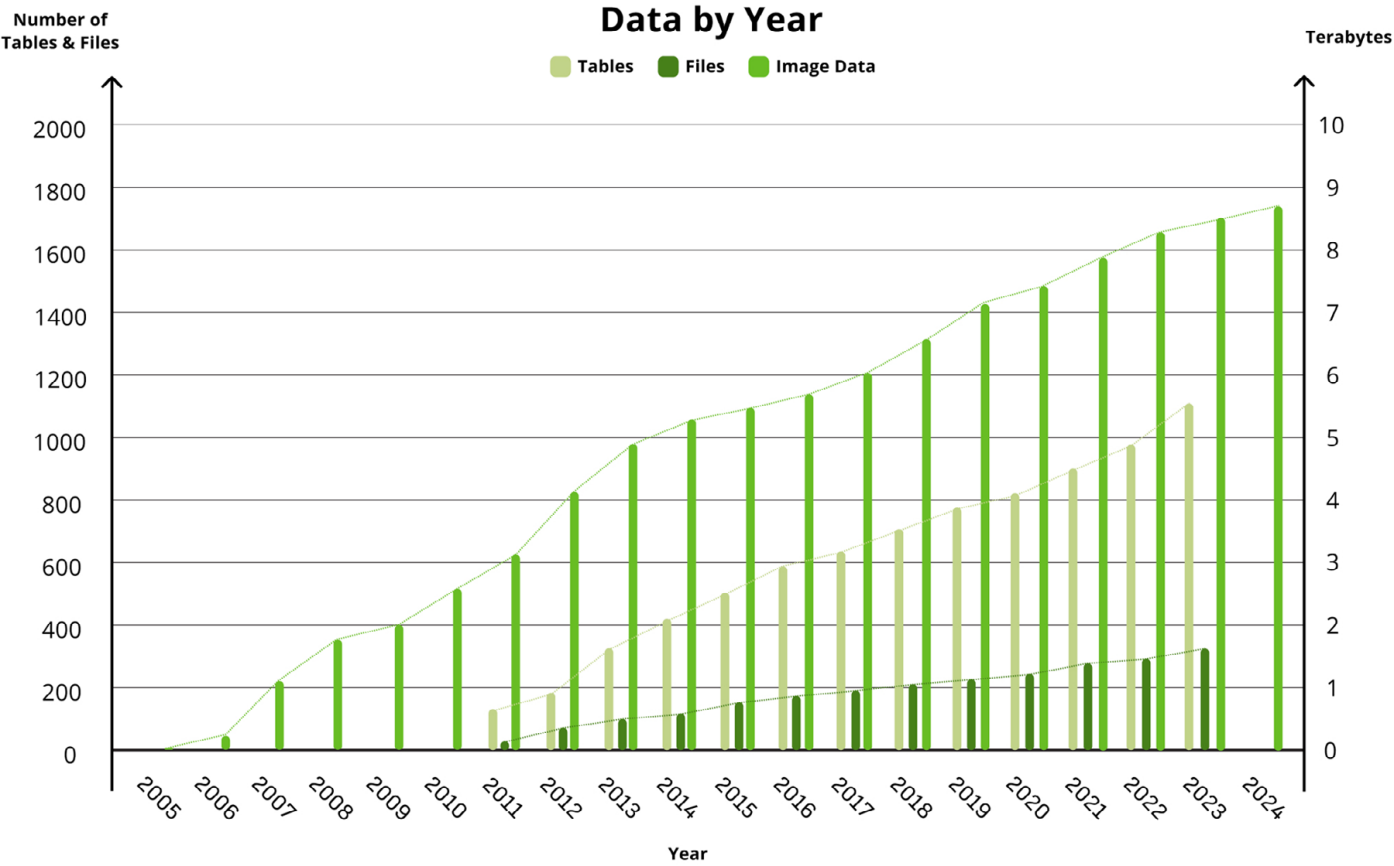
ADNI data added by year. ADNI collects a wide range of data types, including clinical data, genetics data, MRI data, PET image data, and image analysis results, providing a comprehensive resource for researchers. ADNI supports a multimodal approach to Alzheimer's research, integrating genetic, imaging, and cognitive data. ADNI, Alzheimer's Disease Neuroimaging Initiative.

Robust informatics systems are essential to handle the voluminous and diverse data generated across many sites by ADNI's network of researchers, ranging from imaging data to clinical and genetic data. To achieve this, ADNI's Informatics Core established a sophisticated informatics framework at LONI.^4^ ADNI is widely regarded in biomedical research as a gold standard in the generous and timely distribution of high‐quality data, which accelerates research, advances drug development, and ultimately serves the public good.^5^ Data added to ADNI has grown significantly with each passing year (Figure 2).

**FIGURE 3:**
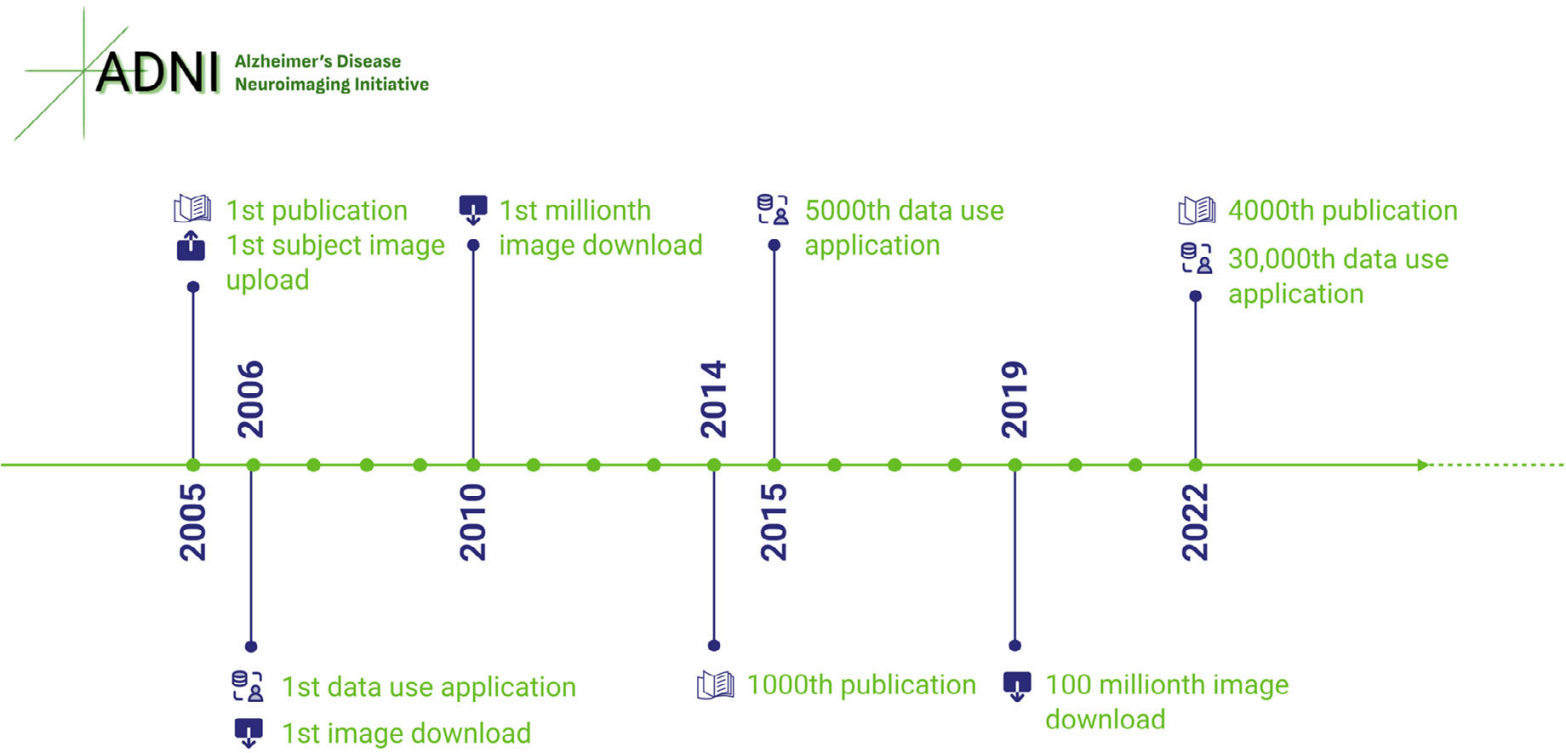
A timeline of ADNI milestones. ADNI has now facilitated over 5583 publications. A total of 185,079 subject images have been uploaded, and 405,414,380 image downloads have been completed. Data use applications have grown to more than 30,000. ADNI, Alzheimer's Disease Neuroimaging Initiative.

The ADNI model has also paved the way for initiatives like the Parkinson's Progression Markers Initiative (PPMI).^6^ PPMI is on a quest to pinpoint biomarkers for Parkinson's disease progression. The ADNI model is also used for the Australian Imaging, Biomarkers and Lifestyle (AIBL),^7^ a longitudinal study investigating biomarkers and psychometric tools to observe AD progression. ADNI similarly paved the way for the Longitudinal Early‐onset AD Study (LEADS),^8^ an observational study to develop sensitive clinical and biomarker measures, and the Advancing Research and Treatment in Frontotemporal Lobar Degeneration‐Longitudinal Evaluation of Familial Frontotemporal Dementia Subjects (ARTFL‐LEFFTDS) Longitudinal Frontotemporal Lobar Degeneration (ALLFTD) research study,^9^ which seeks to learn more about Frontotemporal Lobar Degeneration (FTLD) and understand the difference in brain changes due to FTLD compared to normal aging. Beyond its original scope, ADNI's data have been instrumental in identifying new genetic risk factors for AD.^10^ It have supported research into a wide array of health issues not directly related to AD, highlighting its value as a resource for broader medical inquiries. A timeline of ADNI informatics milestones (Figure 3) depicts significant growth related to ADNI's significance as a resource.

The organization, annotation, and dissemination of data in structured, accessible formats have become indispensable components of scientific research and discovery. Data accumulation is accelerating with the rapid advancement of electronic data collection in clinical, genetic, patient‐reported outcomes, and imaging domains. The imperative to store and share data meaningfully to support analyses, hypothesis testing, and future research endeavors is substantial. However, numerous challenges hinder the realization of value‐added data sharing, particularly when data collection is inconsistent, documentation is inadequate, some level of postprocessing or analysis has already taken place, or coordination among multiple sites is suboptimal.

ADNI is a prospective, defined protocol, coordinated, multi‐site, observational study. ADNI data must first pass quality control (QC) checks, including protocol adherence, before they are made accessible to researchers. Protocols, documentation regarding the data, and methods on how the data were collected are available on the ADNI website.^11^ Comprehensive postprocessing data provenance and the fully described derived data analytic protocols are also available.

## 2 ADNI INFORMATICS CORE

**FIGURE 4:**
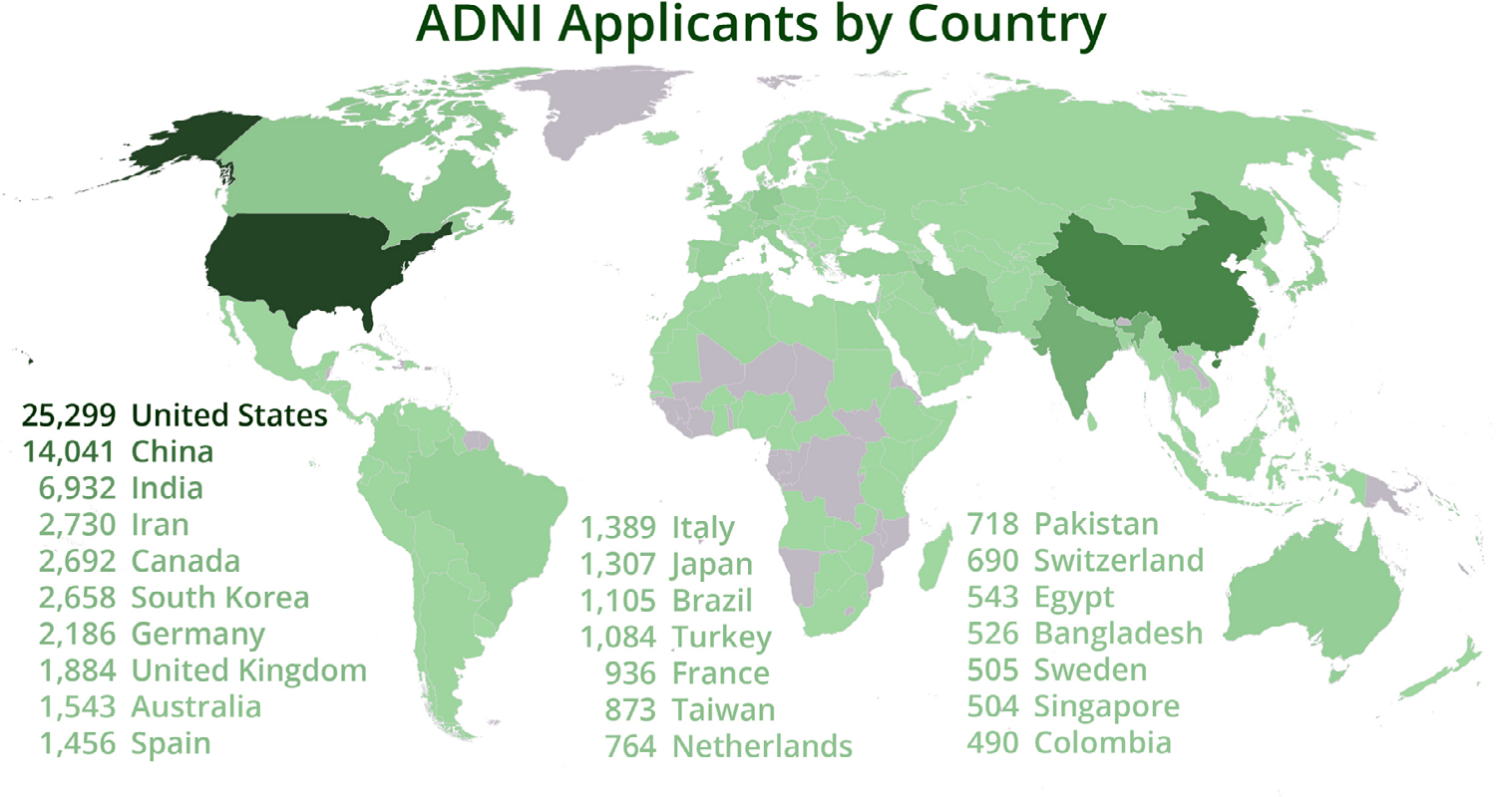
Map of the locations of investigators who applied for data use. ADNI's open data access policy, which provides data without embargo to the entire scientific community, has enabled an extensive global dissemination and utilization of the ADNI data by researchers across academia, industry, and government sectors in numerous countries worldwide. Only the top 25 countries with the highest number of applications are shown and listed. ADNI, Alzheimer's Disease Neuroimaging Initiative.

ADNI comprises eight cores, 59 acquisition sites, and several analysis sites that feed data into the Informatics Core. Data flow through various information systems, institutions, and individuals before being centralized at the informatics repository at LONI. This repository is a centralized and comprehensive informatics hub for well‐described, searchable, and multimodal data, and it provides authenticated researchers access to that data.^4^ Access has been granted to over 26,000 active investigators from 169 countries and has supported more than 405 million downloads of imaging, clinical, biomarker, and genetic data (Figure 4). The ADNI Informatics Core provides a user‐friendly web environment for all information regarding the ADNI project, including the protocols, the overall design and phases of ADNI, data access request forms, a Data Use Agreement (DUA), ADNI publications lists, analyses methods, and links to all manuscripts that have used ADNI data.

The workflow for managing image data in the ADNI project involves several steps to ensure data quality and accessibility. Initially data collected from acquisition sites are entered into clinical and imaging databases. Imaging cores then conduct QC and preprocessing of MR and PET images, followed by postprocessing and analysis by ADNI image analysts. Meanwhile, biofluid samples are processed and the results are compiled. Investigators can then download and analyze the data according to their research needs.

Access to ADNI data is tightly regulated and limited to site users and individuals, as approved by the Data Sharing and Publication Committee (DPC). Access levels determine the system features available to users: acquisition site personnel are restricted to uploading data from their site, whereas imaging core leaders are permitted broader capabilities such as uploading, downloading, editing, and deleting data. All data transactions are meticulously logged for transparency and accountability. Any notification regarding data corrections or recall can be selectively sent to users who have already downloaded those specific records.

The ADNI DPC oversees access for external investigators with an integrated online application and review process. This ensures that applicant information and committee decisions are seamlessly recorded, with automated email notifications for application status. Approved users are required to submit annual progress reports, facilitated by the online system, which also supports related tasks such as adding team members to approved applications and submitting manuscripts for DPC review. Detailed logs track all data accesses, providing study leadership with insights into data use at a granular level, whereas interactive project summary features provide project managers with insights into upload and download activity.

**FIGURE 5:**
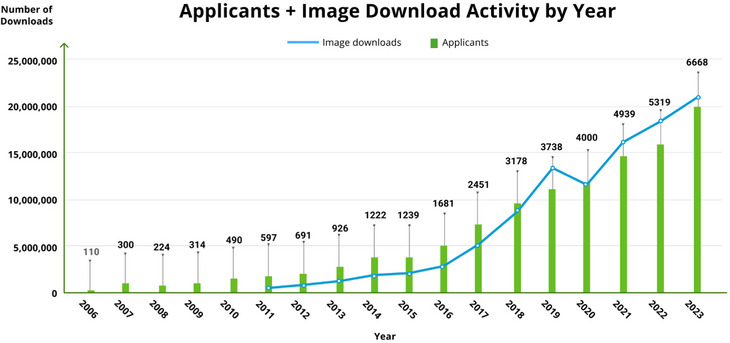
ADNI applicants and image downloads by year. The number of annual image downloads from ADNI has steadily increased over the years, reflecting the growing importance of this data set in AD research. In 2022 alone, there were over 20 million data downloads from the ADNI database, highlighting the scientific community's immense interest in and utilization of this valuable resource. The primary users of ADNI data include academic researchers, pharmaceutical and biotechnology companies, scanner manufacturers, government scientists, and others, including high school teachers. AD, Alzheimer's disease; ADNI, Alzheimer's Disease Neuroimaging Initiative.

**FIGURE 6:**
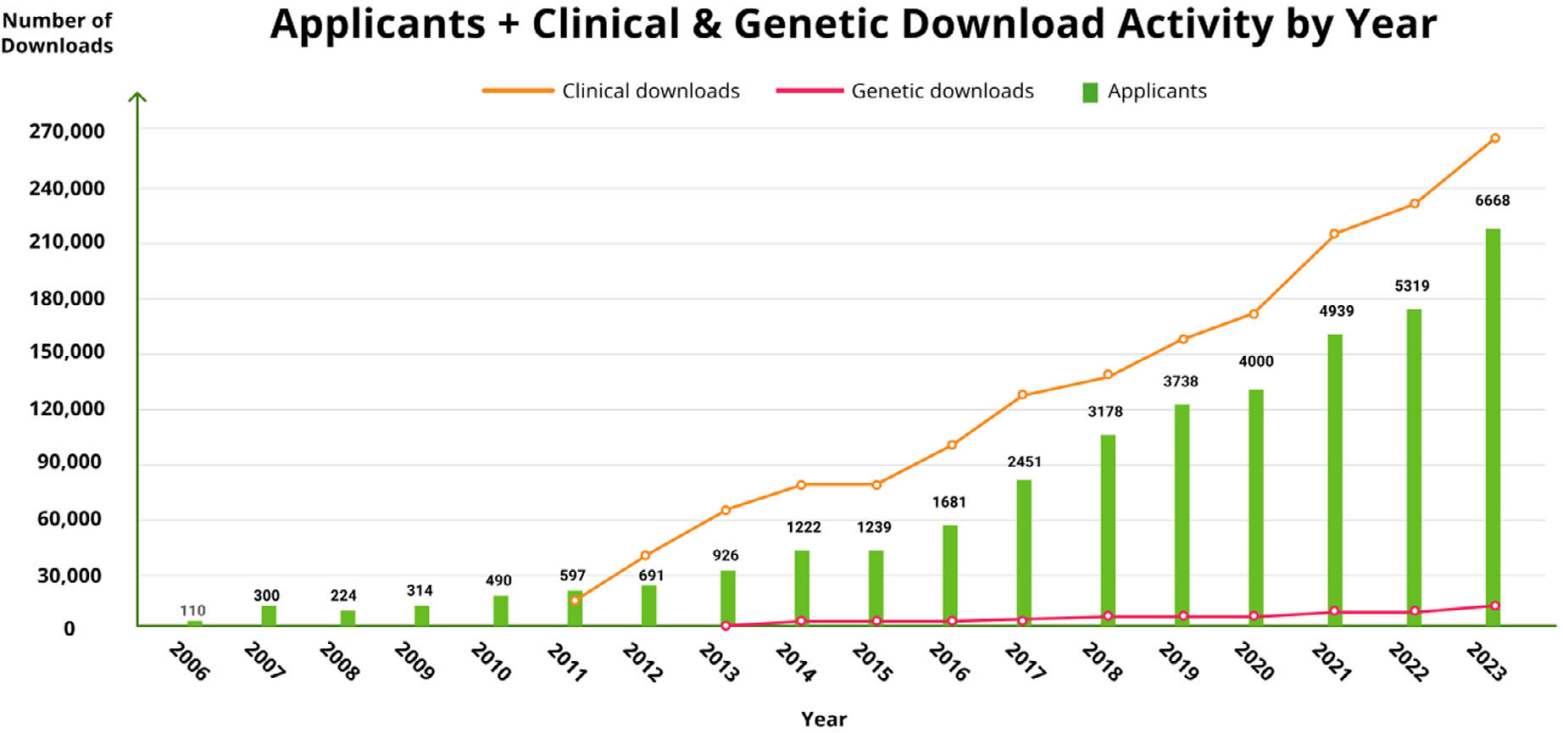
ADNI applicants and clinical and genetic downloads by year. The number of annual clinical and genetic downloads from ADNI has increased over the years, necessitating the ADNI Informatics Core to build and manage a robust and sophisticated informatics framework to meet the growing demand. ADNI, Alzheimer's Disease Neuroimaging Initiative.

The IDA at LONI^12^ was established to aid in data integration, access, and sharing among the growing community involved in neuroscience research, and its use for ADNI has supported the widespread distribution of ADNI data to the scientific community. More than 47,735 total unique user accounts for ADNI have been created over the last 20 years, and 26,588 accounts are currently active (Figure 5). The annual number of applicants for ADNI data has grown exponentially over the last 15 years (Figures 5 and  6). Approved investigators have access to over 108,000 image data sets and related clinical, imaging, biomarker, and genetic data, resulting in more than 105 million downloads of raw and processed scans. Users from around the world have accessed ADNI data continuously and download activity has been steadily increasing every year. The extensive amount of ever‐growing data collected in ADNI, including imaging, clinical, cognitive, biochemical, and genetic information, requires robust systems to process, integrate, and share it. In concert with ADNI changes and expansions over the years, the IDA has evolved to support ADNI data flows, storage, backup, searching, and sharing, with ongoing efforts to enhance data discovery and visualization. Its automated systems manage de‐identification tasks to securely upload and archive data, extract MRI and PET metadata from image files, coordinate data workflows among QC sites, integrate information across data types, and manage, log, and control data access.

### 2.1 Data archive to data hub

The IDA was originally named the “Image Data Archive.” It was designed primarily to de‐identify and upload MRI and PET scans and then make these scans available to researchers to download. In the final step of the upload process, the IDA database is updated with information about each uploaded scan, and researchers utilize IDA search pages to form collections of image scans to download. This image‐centric focus dictated the primary operations of the IDA in the beginning years of ADNI. In the following years, the IDA expanded the types of data it archived as ADNI began to include tabular/comma‐separated values (CSV) data, portable document format (PDF) files, and genetic data. In ADNI‐1, creating and populating image scan queues to provide uploaded image files to ADNI cores for download and quality check became necessary. In ADNI‐2, a Representational State Transfer Application Programming Interface (REST API) was developed to support direct uploads of CSV data into IDA database tables, and other Application Programming Interfaces (APIs) were added to import ADNI data into the LONI Pipeline and to provide ADNI data accessed from web pages outside of the IDA. As the needs of the ADNI study changed, the IDA evolved from an image archive into an image data hub and was subsequently renamed the “Image & Data Archive.”

**FIGURE 7:**
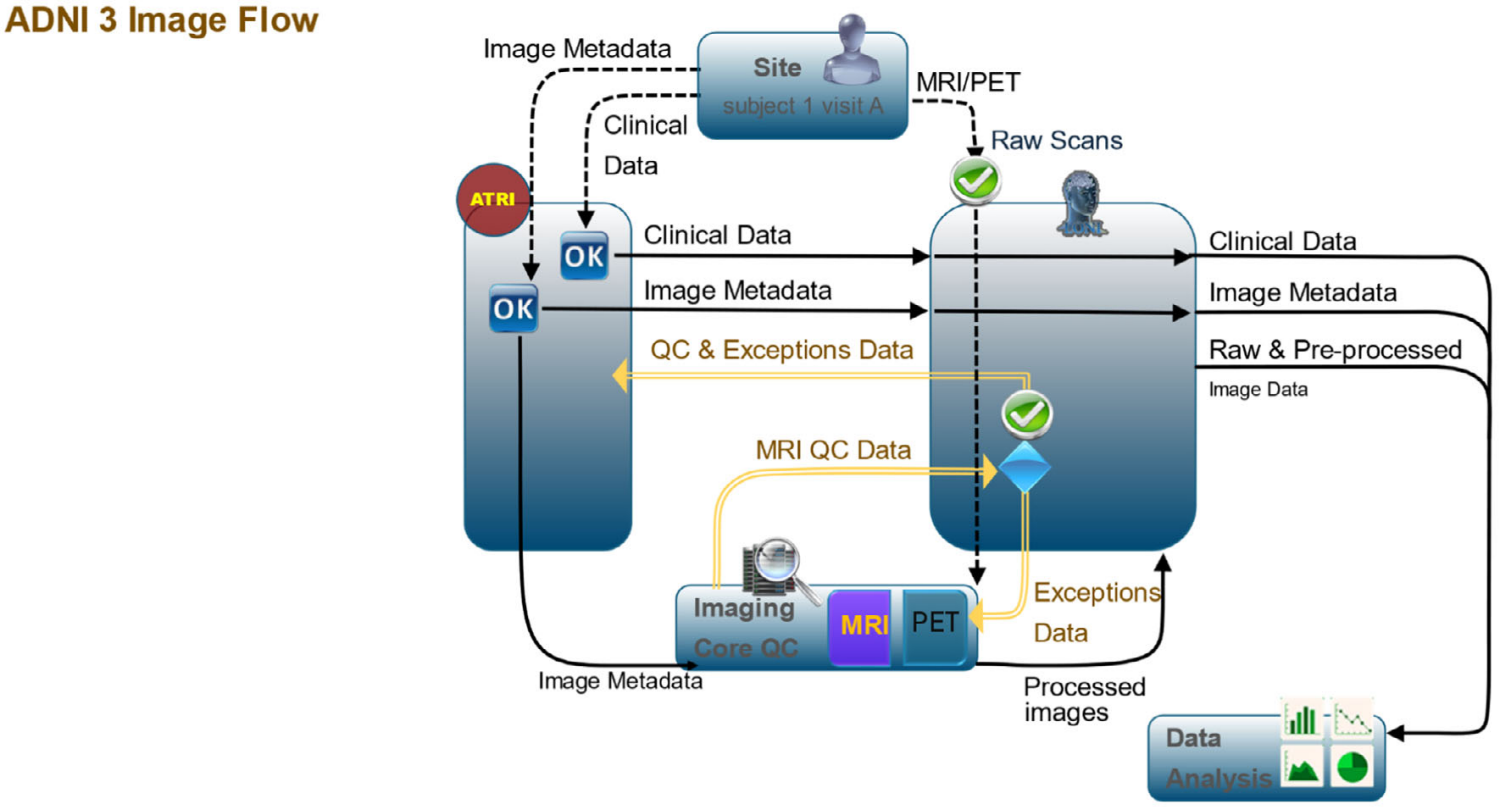
Diagram of the flow between sites, cores, and investigators. ADNI has established a well‐defined data flow process to ensure consistency and quality across the multiple participating sites and cores. ADNI's processes standardize data collection, integrate information across cores, and facilitate data sharing among investigators. ADNI, Alzheimer's Disease Neuroimaging Initiative.

To make data available to the scientific community while meeting the needs of core investigators, a workflow was adopted to upload image data to the repository through the LONI IDA (Figure 7). This process involves validating subject identifiers, removing potentially identifying information, checking image appropriateness (matching to the expected modality, standardized encoding, etc.), encrypting data transmission, and extracting metadata elements for optimal storage and findability. After undergoing quality assessment, the data become accessible to authorized users.

To make processed images standardized across sites and scanners available for analysis, a CSV‐guided upload method is used to link image data with provenance metadata, ensuring clarity on data origin and processing. This upload mechanism allows for the validation of image identifiers and supports uploading large batches of preprocessed images with minimal interaction. This automated process enhances collaboration among analysts and facilitates the sharing of processing protocols.

**FIGURE 8:**
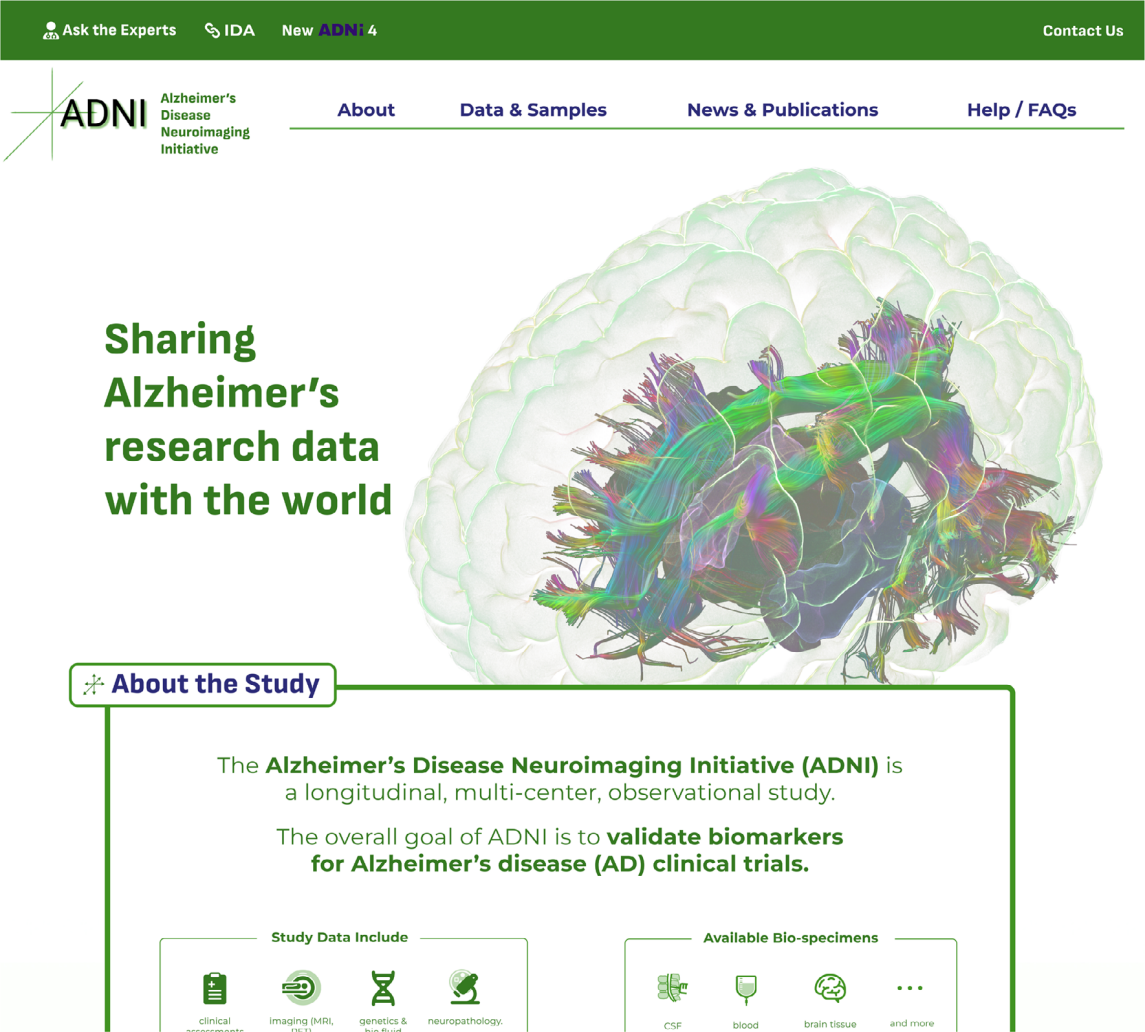
Main page of the ADNI website. ADNI, Alzheimer's Disease Neuroimaging Initiative.

With data oversight and administration dispersed across various institutions, we have developed a suite of tools to assist stakeholders in managing different aspects of the ADNI study. These tools include data user management tools for evaluating data use applications, overseeing manuscript submissions, and sending reminders to investigators about their annual ADNI update. Support for internal cores includes developing and providing reconciliation reports that compare Electronic Data Capture (EDC) data with uploaded image data to ensure that all expected scans have been uploaded and are properly labeled. In addition, we created project summary tools that offer interactive insights into upload and download activities categorized by site, user, and time frame, with the option to export this data for further analysis. Furthermore, our user‐friendly web environment offers a wealth of information, documents, and resources to keep investigators informed about the study's progress and the data available in the archive, thereby enhancing the user experience for researchers worldwide (Figure 8).

The ADNI website also includes an “Ask the Experts” feature, which provides support to users through an online form for submitting questions to the ADNI team. It allows users to submit queries related to ADNI and specific cores, and a response is provided within 3 to 5 business days. The responses are then published in the ADNI Experts Knowledge Base for future reference. In addition, the Informatics Core provides support to investigators via email, fielding hundreds of questions each year.

### 2.2 Infrastructure

To democratize access to ADNI data, the decision was made early on to avoid cloud‐based servers’ “pay as you go” pricing model.. Unlike cloud‐based applications that incur costs for each transaction and frequently pass the costs to the users requesting data, we have instead created an on‐premises infrastructure to receive, store, and distribute files and database tables of ADNI data. The sheer volume of data and number of concurrent users moving vast quantities of data through our environment necessitated the use of high‐performance systems along with a constant upgrade path to keep up with ever‐increasing needs. Each hardware component of our infrastructure is required to provide optimal and reliable data transfer speeds, and our fault‐tolerant network has been designed to maintain high levels of security at each access point. We have redundant servers, databases, load balancers, switches, and firewalls to eliminate single points of failure and distribute computational loads.

The infrastructure initially used to manage ADNI‐1 data consisted of one web server and one database server at University of California, Los Angeles (UCLA). These servers shared resources with other LONI applications and managed files alongside shared directories used by laboratory researchers. However, as ADNI utilization grew, the competition for computational resources and storage space became untenable. In particular, it became apparent that IDA users downloading data were taking resources away from ADNI sites, which were motivated to upload image scans quickly after their acquisition.

The current production system for the IDA at USC is completely segregated from the other computational activities at LONI. IDA network traffic is logically separated at the LONI Palo Alto firewall from all other LONI network traffic, and the database, web server, and development systems each have their own Virtual Local Area Network (VLAN). Upload activities are managed by two redundant servers that are kept separate from two other redundant servers that manage downloads. Redundant load balancers manage all network communications between servers, and security has been increased by adding multi‐factor authentication (MFA) for all administrative logins. In line with our commitment to security and privacy, all data transfers are encrypted using Secure Sockets Layer (SSL) encryption. When possible, we also conduct post‐transfer redundancy checks to safeguard the integrity of our data.

All ADNI data are copied to backup systems to preserve data integrity and protect against catastrophic failure. At the beginning of ADNI‐1, data were written to a StorageTek tape system that used a robotic arm inside a large cylindrical glass enclosure to copy files onto a library of 50 GB tape cartridges that were regularly deposited in an offsite Iron Mountain vault in Colorado. Due to limited storage capacity and speed of writing to each tape, the tapes could reach full capacity before all files were copied. Occasionally, and as the volume of data increased, the daily backups would need more than 24 h to complete, which created problems when the next day's backups were started before the previous day's backups were finished. In ADNI‐2, this backup system was replaced with another robotic system with denser (6.26 TB cartridges) and faster media, and in ADNI‐3, we transitioned entirely to the cloud‐based Amazon AWS S3 Glacier service, which provides a low‐cost solution for storing files offsite with the caveat that it takes over 24 h to retrieve files from backup. Currently, we conduct daily incremental backups of ADNI files and full backups of ADNI database data. In ADNI‐4, which began in 2022, we are migrating our local storage to a solid‐state CEPH cluster consisting of 120 61 TB drives, a subset devoted exclusively to the IDA.

### 2.3 Data management

The IDA image uploader process has always ensured that sensitive metadata and other details, for example, personal health information (PHI), written to image files by scanners are modified or removed before de‐identified image files are uploaded to the IDA. As ADNI began acquiring image data after Health Insurance Portability and Accountability Act (HIPAA) regulations were first enacted, the de‐identification process was designed to conform to its definition of a limited data set, stipulating that certain identifiers must be removed while other protected fields may be disclosed with sufficient obfuscation. Furthermore, the ADNI DUA was written to comply with the privacy regulations stipulated by HIPAA when sharing limited data sets. Multiple de‐identifications are needed for the file formats,  Digital Imaging and Communications in Medicine (DICOM), Emission Computed Axial Tomography (ECAT), High‐Resolution Research Tomography (HRRT), (Neuroimaging Informatics Technology Initiative (NIFTI), and Free Surfer, used by ADNI because each file format defines different metadata fields and stores information in different ways. Although a majority of ADNI MRI and PET scans are written to DICOM files, most data derived from image acquisitions use NIFTI or Free Surfer formats. The de‐identification process run at each ADNI site was first implemented using Java applets executed by web browsers after installing a Java plugin. Although this provided seamless integration with IDA web pages, security concerns eventually led to the deprecation of the Java plugin, and consequently, all major web browsers eventually stopped supporting it.

In 2021, the applets were replaced with installer programs for the Windows, Mac, and Linux operating systems. As security requirements of ADNI sites further increased, corresponding security enhancements were added to digitally sign each installer and remove the former requirement that access be granted to write intermediate files to the local disks of the sites during the de‐identification process. In ADNI‐3, modifications to ADNI de‐identifications occurred when additional requirements were requested (e.g., removal of the patient's birth date in DICOM files). As the number of uploads increased, it also became necessary to provide some uploaders with a command line approach to de‐identify and upload multiple image files sequentially at once—for example, uploads of derived data. Currently this batch process requires creating a CSV file consisting of one row for each upload with columns that define the necessary input data needed to archive the image files.

The total number of image scan files archived in the IDA has grown considerably during the ADNI study, along with their overall size. In ADNI‐1 (2004–2010), image scans requested by IDA users were first written to a ZIP file, and afterward, the ZIP file was made available for downloading. However, as ADNI data grew, this resulted in longer wait times for downloads. By ADNI‐2 (2011–2016), we had changed our download methodology to store archived data in pre‐zipped data chunks that could be assembled as requested into ZIP files. In ADNI‐3 (2017–2022), we incorporated the data chunks into the proprietary file format that the IDA uses to manage bundles of files on disk. These advances not only reduced the amount of disk space needed to store the data but also provided a means by which ZIP files could be dynamically constructed by concatenating pre‐zipped data chunks together directly from archived data.

Unexpectedly, in ADNI‐1, we discovered that ADNI sites had difficulty with local management of their image scans. This became apparent when multiple sites repeatedly uploaded the same image files. It then became necessary to incorporate logic for duplication detection and management of duplicates to prevent the same scan from appearing multiple times in search results. We also adjusted our backup procedures to remove redundant scans. Conversely, although the IDA does keep track of each download for all users and offers the flexibility to download only image scans that a user has not previously downloaded, many researchers will still download the same image scans multiple times, presumedly because it is easier to replace local data files than to update them.

As different types of ADNI data were made available in the IDA and as the number of ADNI collaborators grew, there also became a greater need for data mapping scripts. Data mapping became necessary to update image scan queues, to update database tables after an ADNI collaborator uploaded CSV data, to release data from quarantine after QC scores were made, and to perform administrative functions during nightly automated tasks. In addition, as ADNI progressed through its different phases, data mapping tools were needed to harmonize database tables. In order to meet these needs, we developed IDA Rules, which manages scripts of Structured Query Language (SQL) commands in nested loops along with user‐defined variables that transform data values and update database tables.

### 2.4 Genetic data

In 2012, the Brin Wojcicki Foundation and the Alzheimer's Association funded whole gene sequencing (WGS) of samples from 800 ADNI participants. A total of 58 external hard drives of WGS data were shipped from the Illumina sequencing facility to LONI over the course of 6 months, and the data on each drive were checked for validity and then copied onto the IDA file system. Corrupted drives were replaced by Illumina when they failed validity checks. On a rolling basis and in a round‐robin fashion, validated drives were subsequently shipped to the Genetics Core at the National Institute on Aging Genetics of Alzheimer's Disease Data Storage Site (NIAGADS). After all WGS data had been sent and verified, they were copied onto a high‐capacity external drive, which then became the media for sharing the data with ADNI researchers. Priority was established using a queue, whereby the external drive was shipped to each approved applicant on a first‐come‐first‐served basis. We often encountered difficulties getting the external drive through customs when it was shipped overseas. On more than one occasion the applicants discovered that their institution lacked sufficient data storage. Shipping and local copy times added sufficient overhead and led to months‐long delays. Faster internet speeds and new data transfer technologies have since improved the WGS data sharing process. New large genetics data sets are now typically obtained by the Informatics Core from cloud storage facilities, and the data are provided to ADNI investigators using an Aspera client–server data transfer process that allows simultaneous downloading by multiple investigators with virtually no wait time.

## 3 DISCUSSION

The ADNI Informatics Core serves as a pivotal hub for disseminating data, results, and knowledge, extending well beyond the internal investigators to encompass the global scientific community. This framework supports a vast spectrum of analytical methods and data interpretation techniques. Indeed, the ability to share and distribute information efficiently is a cornerstone of ADNI's achievements. The databases established through this core have made significant contributions not only to AD research but also to studies in other neurological and psychiatric fields. Many ongoing and upcoming projects in these areas are now adopting the innovative systems pioneered by ADNI's Informatics Core. However, there are notable challenges related to data sharing that must be addressed in order for continued success.

### 3.1 Harmonization

The ADNI Informatics Core collects a diverse range of data modalities such as imaging, genomics, clinical data, and electronic health records. Integrating and sharing these heterogeneous, multimodal data sets across different platforms and analysis tools remains a significant technical challenge.^13^ Harmonizing data dictionaries collected across multiple data collection systems is a current focus that provides exponential benefits to investigators.^14^ Sometimes these are simple differences, such as when coded values for the same variable differ, such as 0 = No in one data set versus 1 = No in another data set. Applying mappings to harmonize coded values eliminates the need for each investigator to do so. Complex harmonization issues can typically require expertise to unravel and solve them, and the details should be captured in a way that enables investigators to see how the changes were applied to support the reproducibility of results. Data harmonization is an active area of research, and the Alzheimer's Disease Sequencing Project Phenotype Harmonization Consortium (ADSP‐PHC) program is especially focused on this goal.^15^

### 3.2 Guidelines and mandates

ADNI has addressed data sharing guidelines by establishing a strong ethos of open and unimpeded data sharing, which has been a central element of its success. ADNI's Informatics Core has proactively led the way in adopting policies, procedures, and technologies to overcome challenges related to data sharing, such as concerns over data quality, ownership, and the potential impact on future publications. By openly sharing every piece of data, ADNI enables external replication of results, comprehensive meta‐analyses, and new studies to use existing data sets. This approach has allowed for alternative and contradictory interpretations of results, fostering a culture of peer review and collaboration.^16^

Data sharing mandates are an important step in expanding the breadth and scope of data sharing. There are many complex issues to address if we are to establish an environment that maximizes the impact of data sharing. Despite these challenges, ADNI stands out as a shining example of a successful, openly shared, usable, and efficient database, showcasing the potential for truly global collaborative research. The implementation of shared authorship in publications that utilize ADNI data helped achieve a widely applauded and copied compromise. Most journals accepted this model, whereby *the Alzheimer's Disease Neuroimaging Initiative* was included in the author line, and that was translated into PubMed to include those who lead the ADNI program.

### 3.3 Privacy

The evolution of data privacy has significantly impacted data sharing in scientific research over the past two decades. This evolution is driven by increasing digitization, legal requirements, and ethical standards, all of which influence how researchers collect, share, and utilize data. ADNI's enhanced data protection protocols include advanced encryption methods, secure data storage solutions, and controlled access systems. ADNI's anonymization and pseudonymization techniques are used increasingly to ensure that data can be used and shared without exposing personal identifiers.

The ADNI Informatics Core has implemented rigorous de‐identification protocols where sensitive metadata and other details are altered at the originating institutions before de‐identified image files are uploaded to the IDA. We also sequester certain imaging and clinical data fields. Some data are more sensitive and treated with additional safeguards, such as a new re‐facing procedure to disable the potential for facial recognition software to identify participants from MRI and PET scan data. Voice data or mobility data with geo‐location also presents unique challenges to maintaining anonymity. ADNI will share only obscured versions or analyses of these newer data types and keep the more sensitive versions of them in quarantined and protected archives. These procedures adhere to HIPAA regulations, mandating the excision of specific identifiers while permitting the disclosure of other protected fields, albeit in an obfuscated manner.

The push toward open science encourages sharing of raw research data to increase transparency and reproducibility. ADNI and data repositories similar to it are more prevalent, providing platforms for data sharing that are compliant with privacy regulations. However, privacy concerns can sometimes limit the extent to which data are made open, requiring careful management to balance openness with privacy.

## 4 FUTURE DIRECTIONS

ADNI's quest to accurately diagnose and track AD progression has spurred extensive research into a diverse array of biomarkers. From analyzing molecular signatures in blood plasma and cerebrospinal fluid (CSF) to leveraging cutting‐edge neuroimaging techniques, these approaches offer invaluable glimpses into the biological underpinnings of AD within the brain. Each modality presents unique and often complex challenges to comprehensibly document data, organize them, make them searchable and findable, de‐identify them, and share them.

A good example of this is one of the newest modalities to be collected as part of ADNI‐4: participant voice data. Although plasma, cerebrospinal fluid (CSF), and neuroimaging biomarkers illuminate the pathological changes occurring at a cellular level, voice analysis may capture the functional manifestations of these underlying processes. The large raw data recordings are highly identifying and yet may be sensitive to early consequences of AD and, as such, is an important research topic for ADNI. Although the analyzed results of these recordings are easier to disguise than the raw recordings, the Informatics Core will further develop ways in which these important data can contribute to ADNI without potentially exposing the identity of the participants.

The combination of data exploration and coupled analytics to data archives is the future. Moving huge data files across the internet is not always practical. Coupling compute to the data is a well‐established computer science solution to this problem. We have created model systems with simpler and smaller data archives, such as the Fox Data Exploration Network (Fox DEN),^17^ and with larger and federated systems, like the Global Alzheimer's Association Interactive Network GAAIN.^18^ While these systems have limited analytics attached to them, their potential is clear. Enabling users to explore data prior to downloading is a significant advance.

## CONFLICT OF INTEREST STATEMENT

None of the authors has any conflicts to disclose. The National Institutes of Health supported this effort. Author disclosures are available in the supporting information.

## Supporting information

Supporting Information
